# *Clostridium thermocellum* LL1210 pH homeostasis mechanisms informed by transcriptomics and metabolomics

**DOI:** 10.1186/s13068-018-1095-y

**Published:** 2018-04-05

**Authors:** Jason M. Whitham, Ji-Won Moon, Miguel Rodriguez, Nancy L. Engle, Dawn M. Klingeman, Thomas Rydzak, Malaney M. Abel, Timothy J. Tschaplinski, Adam M. Guss, Steven D. Brown

**Affiliations:** 1 Biosciences Division, Oak Ridge National Laboratory, Oak Ridge, TN USA; 2 BioEnergy Science Center, National Laboratory, Oak Ridge, TN USA; 3 Present Address: Department of Biological Science, University of Calgary, Calgary, AB T2N 1N4 Canada; 4 Present Address: LanzaTech, Inc., Skokie, IL USA

**Keywords:** Proton pumping, F_1_F_0_-ATPase, pH homeostasis, Glutamine synthetase, Glutamate decarboxylase, Urease, GOGAT, Glutamate dehydrogenase, *Clostridium thermocellum*

## Abstract

**Background:**

*Clostridium (Ruminiclostridium) thermocellum* is a model fermentative anaerobic thermophile being studied and engineered for consolidated bioprocessing of lignocellulosic feedstocks into fuels and chemicals. Engineering efforts have resulted in significant improvements in ethanol yields and titers although further advances are required to make the bacterium industry-ready. For instance, fermentations at lower pH could enable co-culturing with microbes that have lower pH optima, augment productivity, and reduce buffering cost. *C. thermocellum* is typically grown at neutral pH, and little is known about its pH limits or pH homeostasis mechanisms. To better understand *C. thermocellum* pH homeostasis we grew strain LL1210 (*C. thermocellum* DSM1313 *Δhpt ΔhydG Δldh Δpfl Δpta*-*ack*), currently the highest ethanol producing strain of *C. thermocellum*, at different pH values in chemostat culture and applied systems biology tools.

**Results:**

*Clostridium thermocellum* LL1210 was found to be growth-limited below pH 6.24 at a dilution rate of 0.1 h^−1^. F_1_F_0_-ATPase gene expression was upregulated while many ATP-utilizing enzymes and pathways were downregulated at pH 6.24. These included most flagella biosynthesis genes, genes for chemotaxis, and other motility-related genes (> 50) as well as sulfate transport and reduction, nitrate transport and nitrogen fixation, and fatty acid biosynthesis genes. Clustering and enrichment of differentially expressed genes at pH values 6.48, pH 6.24 and pH 6.12 (washout conditions) compared to pH 6.98 showed inverse differential expression patterns between the F_1_F_0_-ATPase and genes for other ATP-utilizing enzymes. At and below pH 6.24, amino acids including glutamate and valine; long-chain fatty acids, their iso-counterparts and glycerol conjugates; glycolysis intermediates 3-phosphoglycerate, glucose 6-phosphate, and glucose accumulated intracellularly. Glutamate was 267 times more abundant in cells at pH 6.24 compared to pH 6.98, and intercellular concentration reached 1.8 μmol/g pellet at pH 5.80 (stopped flow).

**Conclusions:**

*Clostridium thermocellum* LL1210 can grow under slightly acidic conditions, similar to limits reported for other strains. This foundational study provides a detailed characterization of a relatively acid-intolerant bacterium and provides genetic targets for strain improvement. Future studies should examine adding gene functions used by more acid-tolerant bacteria for improved pH homeostasis at acidic pH values.

**Electronic supplementary material:**

The online version of this article (10.1186/s13068-018-1095-y) contains supplementary material, which is available to authorized users.

## Background

*Clostridium (Ruminiclostridium) thermocellum* is a model fermentative anaerobic thermophile being studied and engineered for consolidated bioprocessing of lignocellulosic feedstocks into fuels and chemicals [1–3]. *C. thermocellum* forms specialized biofilms for growth on cellulose [4] and produces mobile [5] and cell-bound enzyme complexes termed cellulosomes [6] that mediate deconstruction of lignocellulosic substrates to short, β1,4-linked glucose oligosaccharides for fermentation to ethanol and organic acids. Wild type *C. thermocellum* fermentation products include acetic acid, lactic acid, formic acid, H_2_, ethanol, and amino acids such as valine [7], with additional products being made at high substrate loadings as a result of overflow metabolism [8].

Organic acid production decreases biofuel yields and acidifies culture medium, each of which are undesirable for an industrial process. Therefore, metabolic engineering efforts have focused on modifying carbon metabolism to decrease production of acidic co-products and increase ethanol production [9–17]. In the highest yielding *C. thermocellum* strain published to date, strain LL1210, pathways for the synthesis of lactic acid, acetic acid, formic acid, and most H_2_ production were eliminated [12], followed by adaptive laboratory evolution to improve growth rate and ethanol titer [18]. This allowed the strain to produce 22 g/L ethanol from 60 g/L cellulose, with a maximum theoretical yield for ethanol of 75% although further advances are required for the bacterium to be industry-ready. Ethanol yields and titers of up to 80% and 38 g/L, respectively, have also been achieved with an engineered *C. thermocellum* strain in co-culture with an engineered *Thermoanaerobacterium saccharolyticum* strain [10]. While these strains synthesize essentially no organic acids as end products, fermentation of sugars to reduced compounds such as ethanol or butanol results in the production of a more oxidized compound such as bicarbonate, which will also result in acidification of the medium.

Organisms must be tolerant to the fermentation conditions and the products formed during growth, especially at the high substrate levels required for industrial fermentations. The response to ethanol stress and ethanol-tolerant strains of *C. thermocellum* have been characterized [19, 20]. *C. thermocellum* inhibition by pentose sugars [21] and compounds generated within switchgrass fermentations [22] has also been characterized. However, deep understanding of the *C. thermocellum* response to low pH remains underexplored.

While the anaerobic cellulolytic bacterium *Fibrobacter succinogenes* has been evolved for steady-state growth at pH 5.75 [23], known anaerobic cellulolytic bacteria typically do not grow at pH values lower than pH 6.0 [2]. High-yielding- and highly productive ethanologens such as *Saccharomyces cerevisiae* and *Zymomonas mobilis*, which are not natively cellulolytic, have broad pH ranges (pH 2–6.5 [17] and pH 3.5–7.5 [18], respectively). *C. thermocellum* is typically cultured in medium maintained at neutral pH and it maintains its intercellular pH between ~ 7.3–8.5 [24, 25]. For different *C. thermocellum* strains, growth has been reported between pH 5.9 and 8.1 [26], as low as pH 6.0 [27], and between pH 6.2 and 7.7 [28]. The lower pH limit of the only *C. thermocellum* strain that is currently genetically tractable, strain DSM1313, has not been reported.

A major mechanism of low pH toxicity is dissipation of the proton gradient across the cytoplasmic membrane. Weak acids such as acetic acid become protonated at the low pH in the supernatant, resulting in an uncharged molecule that can cross the cytoplasmic membrane. The cytoplasm is more alkaline, so the weak acid becomes deprotonated, effectively transporting a proton from outside the cell to the inside, collapsing the ∆pH and acidifying the cytoplasm. Cytoplasmic pH can be maintained by a variety of mechanisms that include proton pumps, antiporters, production of ammonia via urease and arginine deiminase pathways, and organic acid decarboxylation reactions that cleave a carboxylic acid side chain (pKa ~ 4.7) to bicarbonate (pKa = 6.4 and more volatile); tolerance to lower pH can be further enhanced by macromolecule repair or protection, lipid changes and biofilm formation [29–31]. Understanding and enhancing the mechanisms by which *C. thermocellum* responds to decreased pH will be critical for the industrial application of *C. thermocellum* for lignocellulosic biofuel production, but those mechanisms are not currently known. Therefore, to better understand the physiological response of *C. thermocellum* to acidic conditions, we cultured strain LL1210 at different pH values in chemostats and applied systems biology tools.

## Results

### *C. thermocellum* LL1210 chemostat growth and lower pH limits in MTC medium

**Fig. 1:**
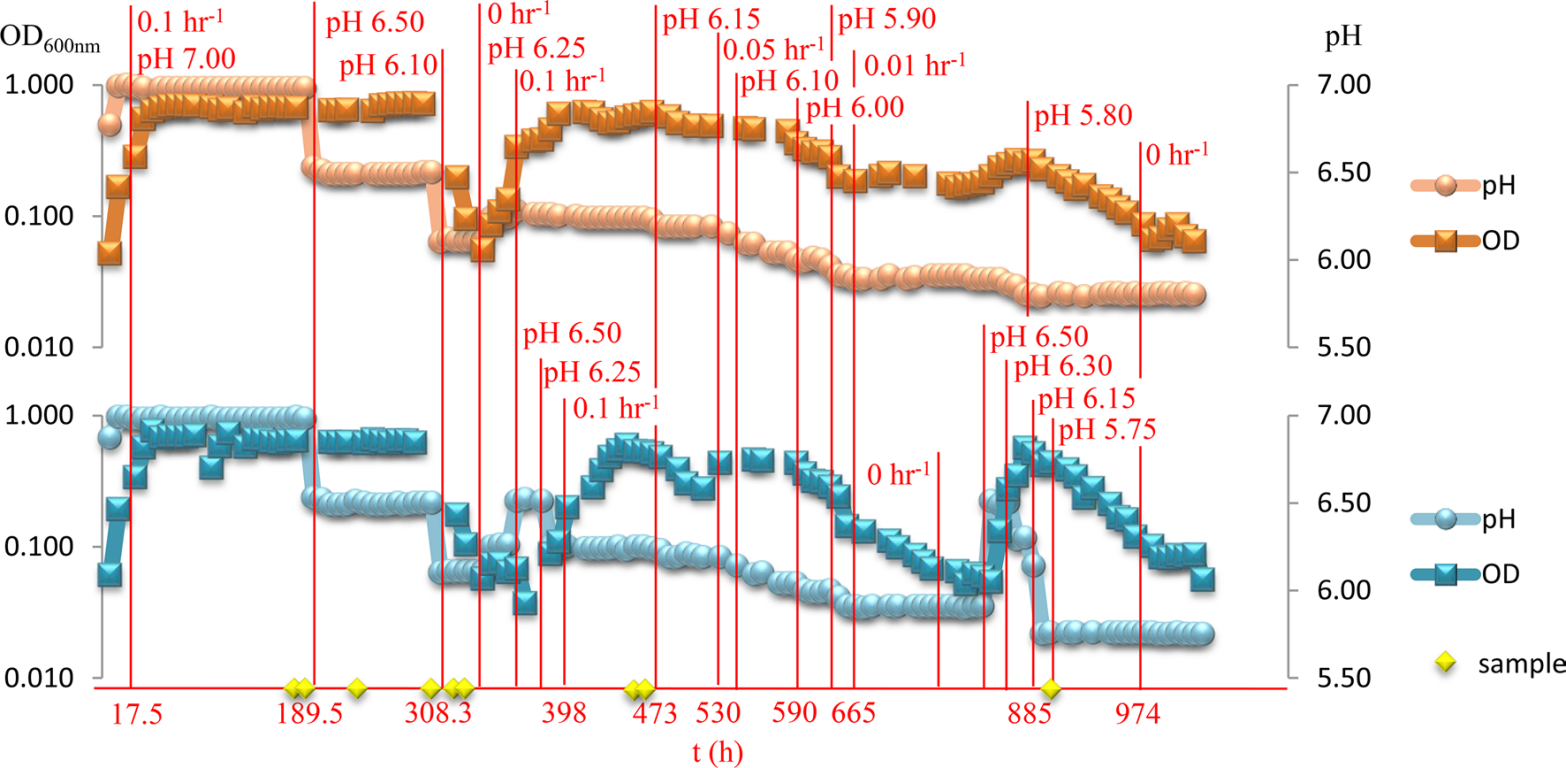
Growth and pH of duplicate chemostat cultures of *Clostridium thermocellum* LL1210. Red lines with red text indicate changes in pH set point and dilution rate for each reactor (orange and blue). pH is represented as spheres and OD as squares. Yellow stars are time points sampled for omics analyses

*Clostridium thermocellum* LL1210 was cultured in duplicate chemostats fed with defined medium (MTC) containing 3 g/L cellobiose for 1058 h (Fig. 1, Additional file 1). After initial batch fermentation, steady-state chemostat growth was achieved for pH 7.0 at a dilution rate of 0.1 h^−1^. The culture pH was then reduced to pH 6.5 at hour 189.5 and stead-state growth was maintained. Culture pH was checked using a regularly calibrated external probe and reported in Additional file 1. A pH adjustment to pH 6.10 resulted in substantial decreases in cell densities (~ 308 h, Fig. 1), along with lower ethanol concentrations and concomitant increases in cellobiose concentrations (Additional file 1), which suggests the specific growth rate of strain LL1210 was lower than the dilution rate of 0.1 h^−1^. Medium flow was temporarily halted for 37.5 h and the culture pH set to 6.25, which restored cellobiose consumption, ethanol production and cell density and permitted medium flow to be resumed at dilution rate of 0.1 h^−1^. After the equivalent of three fermentor vessel volumes, the chemostats were adjusted from pH 6.25 to 6.15, which coincided with lower culture densities. The mean culture turbidity measurements nearest to the omics sampling points, as measured by optical density (OD_600 nm_) and their standard deviations were 0.655 (0.02), 0.651 (0.04), 0.533 (0.06), and 0.143 (0.05) for pH values pH 7, 6.5, 6.25 and 6.1, respectively. A change in dilution rate to 0.05 h^−1^ permitted growth at pH 6.10 and 6.00. At pH 5.90, culture turbidity steadily declined; the dilution rate was reduced to 0.01 h^−1^ and the OD continued to decline for 144 h in one chemostat while the other oscillated, indicative of stress, and eventually recovered. A single chemostat was able to maintain growth at pH 5.90 at a dilution rate of 0.01 h^−1^, although culture turbidity showed a steady decline at pH 5.80 over a 133 h period and was unable to recover when the flow was stopped for 84 h. Thus, growth-limiting pH for this system is approximately pH 6.25 using strain LL1210 in a chemostat with a dilution rate of 0.1 h^−1^ and slower dilution rates are required for steady-state growth below this pH.

Samples at dilution rate of 0.1 h^−1^ were withdrawn from each bioreactor at pH 7 (actual pH = 6.98), pH 6.5 (actual pH = 6.48), pH 6.25 (actual pH = 6.24), and at apparent washout pH 6.15 (actual pH = 6.12) for analysis by transcriptomics and metabolomics (Fig. 1). Additional metabolomics samples were taken at pH 5.80 at a lower dilution rate (0.05 h^−1^) to help elucidate the physiological changes.

### pH homeostasis mechanisms inferred by differential gene expression

**Fig. 2:**
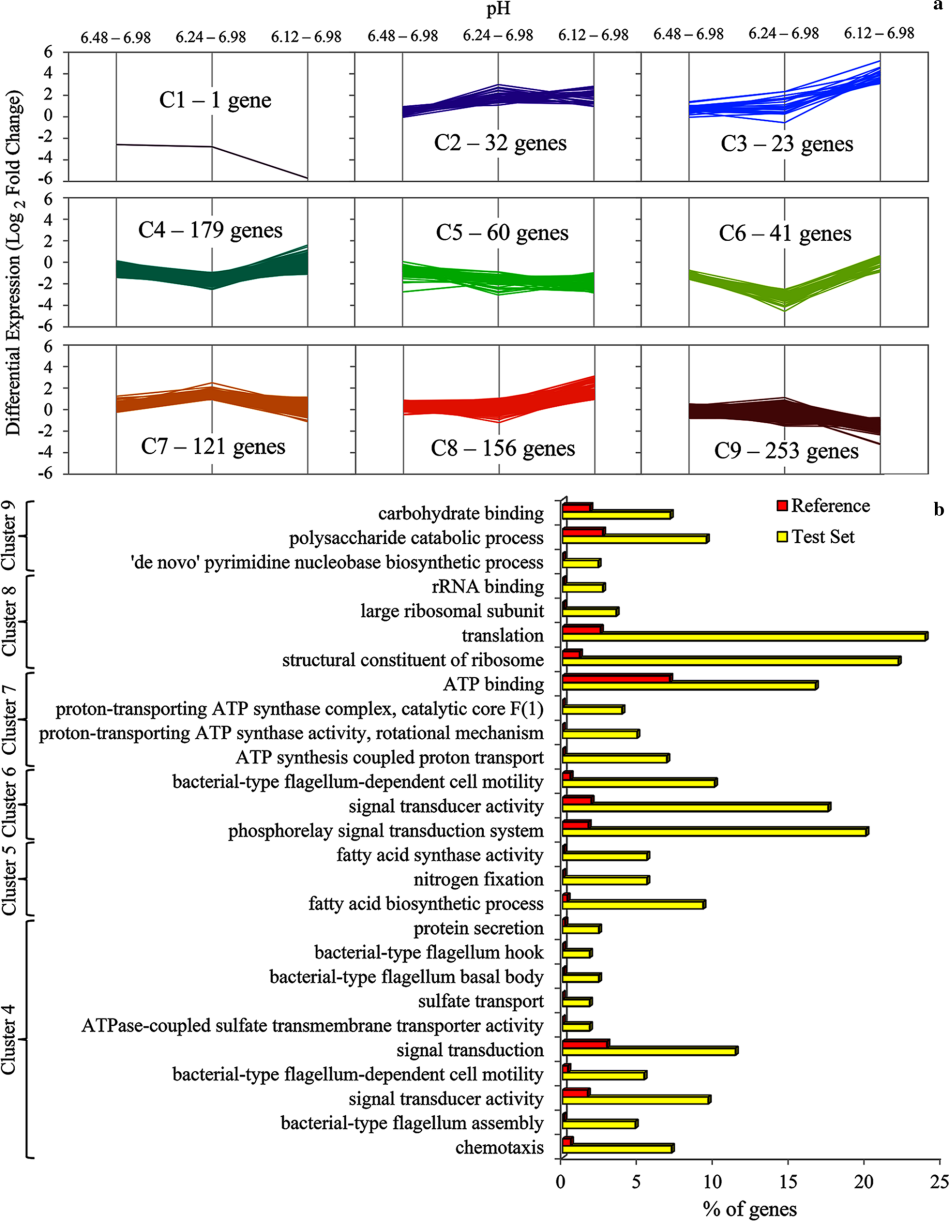
K-means clustering of differentially expressed genes (**a**). GO term enrichment of clustered genes by Fisher’s exact test (**b**). 866 differentially expressed genes from samples taken at pH 6.48, 6.24, and pH 6.12, compared to samples taken at pH 6.98 were clustered. The % of genes represents the percentage of genes within a cluster that contain the given GO term. Enrichment of GO terms indicates that a significantly higher percentage of genes with GO terms are in clusters (Test Set) than in the *C. thermocellum* reference genome (*α* = 0.05)

A greater number of differentially expressed genes were observed as the pH diverged further from pH 6.98. Using pH 6.98 as a reference, there were 80, 469, and 536 differentially expressed genes, respectively, for cells growing at pH 6.48, 6.24, and pH 6.12 (Additional file 2). To more easily categorize gene expression responses to pH changes, differentially expressed genes were clustered (Fig. 2a), and then gene clusters were checked for enrichment of gene ontology (GO) terms in comparison to the frequency of GO terms for all genes in the *C. thermocellum* genome by Fisher’s exact test (Fig. 2b). Cluster 1 contained only one gene (a 2-isopropylmalate synthase; Clo1313_0857) and cluster 3 only contained tRNA genes and were omitted from enriched function tests. Cluster 2 contained genes for rRNA, tRNA, tRNA modification, purine metabolism, and polyamine transport but no enrichment of GO terms was found. Flagella biosynthesis and chemotaxis, sulfate transport and reduction, nitrate transport and nitrogen fixation, glutamate dehydrogenase (GLDH, Clo1313_1847), and fatty acid biosynthesis (clusters 4, 5, 6) were downregulated, while F_0_F_1_-ATP synthase genes (cluster 7) were upregulated at pH 6.24. Diguanylate cyclase genes Clo1313_1339, Clo1313_1404 (with TRP repeats), Clo1313_1813 (with putative polar amino acid sensor domains and a phosphodiesterase domain), and Clo1313_2478 (putative response regulator) were all found in cluster 4. Genes for MotAB (Clo1313_0056-7) were part of cluster 6. MotAB system has been show to generate flagellar torque using proton-motive force [32]. Upon being shocked with pH 6.12, cells increased expression of genes for translational machinery (cluster 8), while gene expression decreased for carbohydrate binding and polysaccharide catabolic processing enzymes (cluster 9) including those for cellulosomal proteins CipA, OlpB, Orf2p, OlpA, CelD, CelH, CelR, CelV, CprA, CtManF, LecA, XynA, and XynD (Additional file 2). Cluster 9 also contained a malate shunt gene, phosphoenolpyruvate carboxykinase (PEPCK, Clo1313_0415) [14]. All but one of the glutamate synthase (GOGAT) genes (Clo1313_2032-3, Clo1313_2035-6) were also in cluster 9.

Previous studies have identified genes commonly expressed during acid stress by Gram-positive neutrophilic bacteria [30], which includes genes for amino acid decarboxylases/antiporter systems, DnaK, GroEL, ClpP protease, urease, F1F0-ATPase, arginine deiminase (ADI), agmatine deiminase (AgDi), and cyclopropane fatty acid (CFA) synthase. Most of these gene systems were not differentially expressed in *C. thermocellum* and genes for ADI, AgDi, or CFA are not annotated in the genome. Rather than increasing in expression at low pH, several decarboxylases were downregulated at pH 6.24 and 6.12, including aspartate 1-decarboxylase (Clo1313_1318), diaminopimelate decarboxylase (Clo1313_1540), orotidine 5′-phosphate decarboxylase (Clo1313_1266), putative oxaloacetate decarboxylase (Clo1313_1523), and a putative sodium pump decarboxylase γ-subunit (Clo1313_1525). The only decarboxylase that was upregulated at any pH was the *S*-adenosylmethionine decarboxylase proenzyme (Clo1313_1509), which produces the aminopropyl substrate need for synthesis of the polyamines spermidine and spermine. However, expression of the agmatinase and spermidine synthase (Clo1313_1529-1530) that synthesize the spermidine and spermine were either not differentially expressed or showed decreased expression at lower pH. Then again, genes for a putative spermidine/putrescine ABC transporter (Clo1313_1471-5) were upregulated at pH 6.24 and 6.12 (Additional file 2). Because a polyamine transporter and biosynthetic gene were upregulated, and polyamines can decrease membrane permeability and may protect cells in acid environments [33], their potential to augment *C. thermocellum* LL1210 growth at lower pH values was evaluated. Medium was supplemented with 100 μM of the polyamines putrescine, spermidine, spermine, or metabolic precursor arginine and the terminal pH was determined after 144 h. *C. thermocellum* LL1210 fermentations with and without amendments initiated at pH 7 and 6.75 had similar growth profiles (Additional file 3).

Downregulation of the GLDH (Clo1313_1847) and GOGAT (Clo1313_2032-3, Clo1313_2035-6) genes were observed at pH 6.12, but no significant expression changes were observed for the Type I glutamine synthetase (GS, Clo1313_2031), two Type III glutamine synthetases (Clo1313_1357 and Clo1313_2038), or the glutamate synthetase (Clo1313_1849) (Additional file 2). The type III glutamine synthase (Clo1313_2303) was significantly upregulated at pH 6.24 (Additional file 2). To examine the role of nitrogen metabolism in relation to pH and physiology, deletion strains for GLDH, GOGAT, GS, GS–GOGAT, and an annotated NifH (Clo1313_2339) were characterized using unbuffered (MOPS free) carbon-replete medium (10 g/L) (Additional file 4). Consistent with an earlier study [18], the parental strain had a higher specific growth rate compared to strain LL1210 (Additional file 4a). The final pH for the GS-mutant- and LL1210 cultures were about pH 6.3 and pH 5.9, respectively, whereas the range for the other strains was ~ pH 5.6–5.8 (Additional file 4b). In the unbuffered system, the GS mutant achieved the highest culture turbidity and produced the second highest titer of ethanol after strain LL1210 (2.0 and 2.7 g/L, respectively) (Additional file 4c). The higher final ethanol titers and pH values for strains GS and LL1210 coincided lower levels of residual substrate.

Genes for two orphan histidine kinases previously predicted to be part of the sporulation cascade in *C. thermocellum* [34], a pro-σ^E^ processing protease and BofA, inhibitor of the stage IV pro-σ^K^ processing protease SpoIVFB, were upregulated in *C. thermocellum* LL1210 at pH 6.24 compared to 6.98 (Additional file 5). To better understand the role of sporulation in the pH response of *C. thermcellum*, growth and sporulation of strains LL1210, asporogenous strains M1726 (DSM1313 ∆*hpt* ∆*spo0A1*) and M1725 (DSM1313 ∆*hpt* ∆*spo0A1* ∆*ldh* ∆*pta*), and the DSM1313 ∆*hpt* parental strain were examined. *C. thermocellum* sporulation was inefficient, consistent with an earlier study [35] and sporulation-deficient mutant strains had neither a growth advantage or disadvantage at lower pH values for the conditions used in this study (Additional file 5).

The molecular chaperone GrpE (Clo1313_0932) and heat shock protein Hsp 20 (Clo1313_0678) were upregulated (2.0- and 2.2-fold) at pH 6.12, which were previously found to be upregulated after furfural shock and heat shock in *C. thermocellum* ATCC 27405 [36]. Hsp33 (Clo1313_2544) and MutS (Clo1313_1982) were upregulated at pH 6.24 (2.2- and 2.0-fold, respectively). Hsp33 was previously found to be more highly expressed in cellulose-adhered *C. thermocellum* ATCC 27405 compared to planktonic cells [4]. At pH 6.12, downregulated genes included one of the annotated Clp protease genes (Clo1313_1116) (0.48-fold), an annotated DNA repair proteins RadC (Clo1313_1465) (0.36-fold), and UvrD (Clo1313_2004) (0.31-fold) and a small subunit of an exodeoxyribonuclease VII (Clo1313_1389) (0.50-fold).

### Metabolite profiles for low pH conditions

To further investigate the physiological changes that occur at lower pH values, metabolomics was performed to compare intercellular concentrations of metabolites when cells were exposed to pH 6.98, 6.48, 6.24, 6.12, and an additional time point at pH 5.80 (at a lower dilution rate 0.05 h^−1^). Peak areas for 91 metabolites identified by GS–MS were calculated for comparison (Additional file 6) and 39 of these were significantly higher at one or more of the pH values compared to pH 6.98 (Table 1). Most significantly, higher metabolites in cells at pH 6.24 were nitrogen (N)-containing metabolites that included glutamate, lysine, glycine, and five unknown N-metabolites (Table 1). There were also two unknown phosphate (P)-containing metabolites. Glutamate showed the largest differences in average fold change across all samples tested at lower pH values (Table 1). At pH 6.24, cells contained 267 times more glutamate than cells at pH 6.98 (significant *α* = 0.05). Intracellular concentrations of glutamate ranged from 0.004 μmol/g pellet at pH 6.98 to 1.798 μmol/g pellet at pH 5.80 (Additional file 6). Concentrations of intercellular valine, alanine, and threonine were also quantified on a μmol/g of pellet basis for comparison with glutamate concentrations. While glutamate and alanine peaked at pH 5.80 (2.660 μmol/g of pellet for alanine), valine and threonine peaked at 6.24 with concentrations of 5.312 and 0.267 μmol/g of pellet, respectively. Long-chain fatty acids, their iso-counterparts and glycerol conjugates accumulated at growth-limiting pH values, pH 6.12 and 5.80 (Table 1). Citramalate accumulated at pH 6.24, 6.12, and 5.80 (Table 1) and an accumulation of glycolysis intermediates 3-phosphoglycerate, glucose 6-phosphate, and glucose at pH values 6.12 and 5.80 was consistent with downregulation of genes for major downstream pathways, malate shunt (Clo1313_0415) and the TCA cycle (Clo1313_0708-9) (Additional file 2).

**Table 1:** Intercellular metabolites that were significantly higher or lower in concentration at below-standard pH values

Metabolite [retention time (min); key m/z]	pH 6.48 vs 6.98		pH 6.24 vs 6.98^α^		pH 6.12 vs 6.98		pH 5.80 vs 6.98	
	Fold change	*p* value	Fold change	*p* value	Fold change	*p* value	Fold change	*p* value
Glutamic acid	57.5	0.429	267*	0.003	319	0.422	471	0.334
10.34 256 156 358 373 N-metabolite	30.4	0.432	83.3*	0.025	28	0.411	57.7*	0.014
3-Phosphoglyceric acid	1.98	0.052	45.2	0.384	10.8*	0.027	46.5	0.374
11.93 288 198 172 390 M+ N-metabolite	2.4	0.140	33*	0.000	14.1	0.194	6.17	0.291
Valine	4.75	0.127	16.7	0.082	4	0.118	12.4*	0.003
Mannose 6-phosphate	1.47*	0.020	14.1	0.364	3.33*	0.008	6.58	0.131
Adenine	2.68	0.509	11	0.122	4.83	0.463	16.6*	0.011
9.76 174 276 186 248 N-metabolite	2.53	0.414	9.21*	0.038	10.4	0.414	17.7	0.251
Alanine	1.87	0.194	8.86	0.145	6.16	0.202	11.3*	0.010
Monostearin	1.55	0.437	8	0.075	1.65*	0.040	15.5*	0.007
Stearic acid	1.84	0.173	7.96	0.174	2.26*	0.023	27.1	0.086
Glucose 6-phosphate	1.62	0.244	7.83	0.253	8.4*	0.004	9.3	0.128
10.81 218 202 320 100 N-metabolite	2.9	0.061	7.5	0.076	1.81	0.354	7.64*	0.024
13.30 299 315 357 328 211 P-metabolite	1.35	0.081	7.36*	0.017	7.52	0.120	29.6	0.088
15.23 218 203 244 N-metabolite	1.98	0.414	7.09*	0.017	2.58*	0.017	14.4	0.062
Lysine	1.74	0.496	6.67*	0.007	9.3	0.393	20.1	0.084
8.92 259 274 184	2.24	0.032	5.08	0.303	2.33*	0.009	8.27	0.141
15.2 347 glycoside	3.25	0.213	4.71	0.391	0.352**	0.006	1.18	0.174
6.96 245 260 102 organic acid	1.8	0.638	4.54	0.057	2.21	0.532	4.37*	0.013
Threonine	1.4	0.155	4.46	0.051	2.33*	0.038	1.52	0.100
Palmitic acid	2.03	0.139	4.17	0.305	2.54*	0.027	11.8*	0.001
Azelaic acid	1.15	0.573	3.33	0.368	1.55*	0.024	4.64	0.175
Isostearic acid	1.08	0.516	3.32	0.237	1.15	0.242	7.18*	0.015
10.89 218 191 100 362	2.5*	0.012	3.23	0.130	0.87	0.501	2.75*	0.028
11.33 299 211 328 415 175 P-metabolite	0.679	0.231	3.1*	0.026	3.09*	0.004	0.885	0.782
Glycine	1.53	0.391	2.67*	0.046	1.52	0.256	2.56	0.226
Isopalmitic acid	1.43	0.144	2.53	0.379	1.55	0.073	6.14*	0.033
Thymine	1.05	0.921	2.42	0.291	1.82	0.196	2.73*	0.036
Phosphate	1.28	0.451	2.21	0.158	1.73	0.125	3.02*	0.013
Citramalic acid	0.864	0.645	2.03	0.092	1.92*	0.041	2.9*	0.008
7.50 216 231 188 172 N-metabolite	1.73	0.524	2.01*	0.031	1.4	0.594	2.46	0.135
Glycerol	0.664	0.007	1.77	0.284	1.43*	0.037	2.86	0.241
Isoheptadecanoic acid	1.11	0.689	1.65	0.537	1.2	0.472	2.94*	0.020
Glucose	1.04	0.791	1.29	0.697	1.38*	0.021	2.79	0.215
Monopalmitin	0.879	0.774	1.2	0.710	1.6	0.107	3.87*	0.025
5′-Adenosine monophosphate	0.29**	0.009	0.644	0.589	2.72	0.364	0.531	0.349
2-Ethylbutylamine	0.591	0.072	0.57	0.058	0.4**	0.038	0.715	0.278
7.23 117 74	0.726	0.642	0.321**	0.015	0.835	0.708	0.489	0.071
15.90 292 uronic acid conjugate	0.268**	0.028	0.0691**	0.022	1.06	0.808	1.15	0.907

^α^Table is ordered by descending fold change of intercellular metabolites at pH 6.24 compared to pH 6.98

* Significantly higher at *α* = 0.05; ** significantly lower at *α* = 0.05

### Extracellular amino acid accumulation at growth-limiting pH

At pH 6.24, only valine, alanine, and threonine extracellular concentrations were significantly higher than when pH was 6.98 (*α* = 0.05) (Additional file 7). Asparagine was significantly lower at pH 6.24 compared to 6.98 (*α* = 0.05). Extracellular glutamate was not significantly different at pH 6.24 compared to 6.98, although it was significantly lower at pH 6.48 compared to 6.24 (*α* = 0.05). When cells were shocked with pH 6.12 compared to when they were growing at pH 6.98, there were significantly lower concentrations of all detectable extracellular amino acids except methionine (*α* = 0.05). The amino acids that had the highest average extracellular concentrations in chemostats were valine (1.5 mM at pH 6.24), asparagine (147 μM at pH 6.98), and alanine (104 μM at pH 6.24). The highest average concentration of glutamate was 32 μM (near the limit of detection) at pH 6.24 (Additional file 7). An ABC-type branch-chain amino acid transporter gene (Clo1313_0822) was upregulated at pH 6.24 and 6.12 and ABC-type polar amino acid transport genes (Clo1313_0794-5) were upregulated at pH 6.24 (Additional file 2).

### Metabolic inhibition at a cellular level

**Fig. 3:**
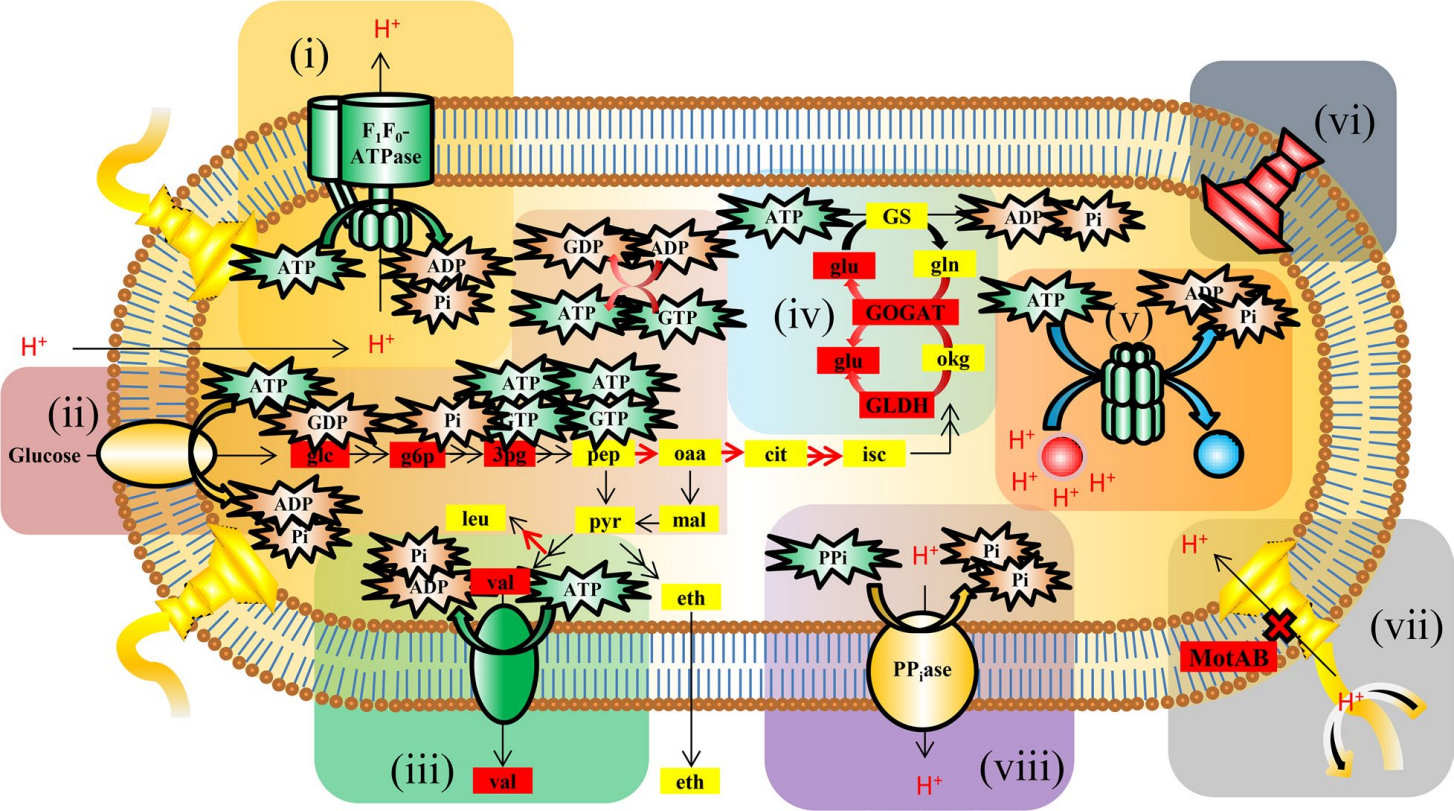
Metabolic imbalances and key expression differences at growth-limiting pH values. A proton-pumping F_0_F_1_-ATPase maintains ∆pH (i). Glycolysis intermediates accumulate indicating inhibition of flux (ii). Valine transport (iii) and glutamine synthetase activity (iv) and chaperones that protect cytoplasmic macromolecules (v) consume ATP. ATP is preserved directly via downregulation of flagella biosynthesis and motility and other ATP-utilizing functions (vi), and indirectly by downregulation of proton-channeling motility genes MotAB (vii). ATP-independent PP_i_ase proton pump (viii). Dark green and red proteins are ones whose genes were up and downregulated, respectively. Dark red metabolites were ones that accumulated

To gain a global perspective on metabolic inhibition at growth-limiting pH values, at and below pH 6.24, key mechanisms and responses for pH homeostasis were summarized (Fig. 3). A key feature of the physiological response at lower pH values was changes in expression for use and conservation of ATP. At pH 6.24, LL1210 increased expression of its F_1_F_0_-ATPase (Fig. 2). The F_1_F_0_-ATPase likely pumps protons to maintain pH homeostasis by ATP hydrolysis as is the case of many other neutrophiles [30]. Competing uses for ATP included valine transport, chaperones protecting cytoplasmic macromolecules from acid damage, and assimilation of ammonium by GS, GOGAT, and GLDH (Table 1, Additional file 2). ATP is also consumed by fatty acid biosynthesis (Table 1, Fig. 2), but there are many metabolites and downregulated genes for this aspect of metabolism. Therefore, it was omitted from Fig. 3, with the exception of biosynthesis pathways for valine and leucine, which are precursors for biosynthesis of iso-fatty acids. The accumulation of fatty acids and iso- derivatives may improve the capacity cytoplasmic membrane to repel protons, but adjustments to membrane lipid composition in response to an increase in proton concentrations are diverse among bacteria and not well understood [37]. Though fatty acid adjustments may have reduced membrane permeability to protons, downregulation of fatty acid biosynthesis genes suggests that ATP conservation was more important. Other gene expression changes that decrease ATP consumption include downregulation of sulfate and nitrate ABC transporter and reduction genes and 80 putative flagella biosynthesis and motility genes (listed in Additional file 2). Reduction of proton influx may also have been achieved through downregulation of gene expression for proton-channeling motility proteins MotAB (Clo1313_0056-7) at pH 6.24 (Fig. 2) [32]. The putative proton-pumping PP_i_-ase (Clo1313_0823) was constitutively and highly expressed at all pH values. Accumulation of intercellular phosphate at pH 5.80 (Table 1) may have been due to PP_i_-ase activity, ATP hydrolysis or other cellular activities. Another possibility is that collapse of the membrane gradient would have made PP_i_ hydrolysis to free phosphate much more thermodynamically favorable via the membrane-bound PPi-ase. Both reduction of proton influx through MotAB and proton-pumping through the putative PP_i_-ase would allow more ATP conservation.

## Discussion

*Clostridium thermocellum* strain LL1210 has deletions in key genes for metabolic pathways to produce acetate, lactate, formate, and H_2_, and it has among the highest reported ethanol yields and titers for this bacterium. LL1210 grew at pH 6.24 at a dilution rate of 0.1 h^−1^. At pH values at or below 6.24, we observed gene expression patterns and metabolite levels consistent with five pH homeostasis mechanisms used by *C. thermocellum* LL1210, namely (i) proton pumping by F_1_F_0_-ATPase, (ii) macromolecule protection by chaperones, and (iii) prevention of proton influx through MotAB were mechanisms supported by changes in gene expression (Fig. 2, Additional file 2); (iv) proton pumping by PP_i_-ases was supported by metabolomics (Table 1); and (v) changes in membrane fatty acid composition was supported by both techniques (Fig. 2, Additional file 2, Table 1).

Differential expression analysis at pH 6.24 revealed that the F_1_F_0_-ATPase genes were upregulated (Fig. 2), consistent with a model that the bacterium uses F_1_F_0_-ATPases to pump-protons at the expense of ATP (Fig. 3). Increased expression and activity of F_1_F_0_-ATPases are associated with pH tolerance in several other bacteria challenged with sublethal pH conditions [29, 30, 38]. In addition to the F_1_F_0_-ATPase system, proton-pumping may be possible via a constitutively and highly expressed putative proton-pumping PP_i_-ase (Clo1313_0823) (top 9% of genes) [39, 40]. *Rhodospirillum rubrum* and plant proton-pumping PP_i_-ases are reversible [41] and the *C. thermocellum* proton-pumping PP_i_-ase has been proposed to be important in energy metabolism in this organism [42]; further studies are required to experimentally test the function in *C. thermocellum*. Many ATP-utilizing enzymes and pathways were downregulated at lower pH, which included most of the flagella biosynthesis genes, genes for chemotaxis, and other motility-related genes (> 50) as well as sulfate transport and reduction, nitrate transport and nitrogen fixation, and fatty acid biosynthesis genes (Fig. 2, Additional file 2). Genes for sulfate transport/reduction and for nitrogen metabolism have shown differential expression in response to stresses [36, 52] and their roles in maintaining homeostasis and for channeling electrons to augment biofuels productively could better understood through modeling, biochemistry and genetic studies. In this study, clustered and enriched gene ontologies (GO) analyses indicate genes for enzymes using ATP had inverse differential expression patterns compared to F_1_F_0_-ATPase genes, which suggests metabolism is redirected to conserve energy (Fig. 2). Indeed, maintaining ∆pH and protecting macromolecules takes precedence over many reactions since critical enzymes for growth cannot function if they are damaged. In addition to a direct burden on ATP, flagella motility may have an indirect burden since protons channeled into the cell through MotAB would need to be pumped back out by to maintain pH homeostasis. Therefore, *motAB* downregulation at pH 6.24 would support pH homeostasis (Additional file 2, Fig. 3).

Genes involved in polyamine transport and biosynthesis were differentially expressed at lower pH, suggesting a potential functional role for these compounds in pH tolerance. Specifically, the transporter (Clo1313_1471-5) and SAM decarboxylase gene (Clo1313_1509) were upregulated (Additional file 2). Medium supplementation with polyamines or arginine (a polyamine precursor) did not significantly improve growth or enable *C. thermocellum* to ferment to a lower terminal pH value compared to unsupplemented fermentations (Additional file 3). Instead, a functional role for stabilization of tRNAs during translation [43] matches well with polyamine biosynthesis and transport gene expression profiles clustering with expression profiles of genes for tRNAs, tRNA modification proteins, rRNA, ribosomal proteins, ribosomal maturation factors, and translational initiation factors (Fig. 2, Additional file 2). Upregulation of translational machinery, tRNAs, and the polyamines that stabilize them, is likely a regulatory response to the abundance of available carbon at pH-limited growth.

Genes involved in sporulation were upregulated at pH 6.24 in chemostat culture. Interestingly, strain LL1210 has a mutation in one of the two copies of the *spo0A* gene that results in an amino acid change [18]; Spo0A is generally the master regulator of sporulation, and the role of this mutation has not been established. However, examination of sporulation in different genetic backgrounds at acidic pH fermentation demonstrated that *C. thermocellum* is inefficient at sporulation, and that LL1210 is similar to the wild type strain. Further, a growth advantage for sporulation mutants in acidic, MOPS-free, carbon-replete medium was not observed (Additional file 5), suggesting that a programmed metabolic shutdown via sporulation is not a major driver of sensitivity to low pH.

Nitrogen metabolism was strongly effected at lower pH values, resulting in increased secretion of amino acids including valine and alanine, which has been seen in other studies [8, 18], and also intracellular accumulation of glutamate with minimal secretion (Table 1, Additional files 5, 7). Some neutrophilic bacteria express glutamate decarboxylase in response to pH stress to convert glutamate into γ-aminobutyric acid; the decarboxylation reaction consumes protons from the cytoplasm which reduces intercellular pH [30]. Heterologous expression of a glutamate decarboxylase in *C. thermocellum* LL1210 could make use of the accumulating intracellular glutamate, possibly enabling steady-state growth at pH values less than 6.25 with a dilution rate of 0.1 h^−1^. Glutamate decarboxylases with pH and temperature ranges suitable for *C. thermocellum* have already been characterized [44, 45], and heterologous expression of glutamate decarboxylase genes with enhanced production of γ-aminobutyric acid in distantly related organisms has been demonstrated [46]. Urease is another target for improvement of pH tolerance in *C. thermocellum*. Urease activity in neutrophilic bacteria produces ammonia which is protonated intercellularly or exported and protonated to form ammonium thereby raising intracellular or extracellular pH [30]. The urease genes had low expression in LL1210 at all pH values sampled (Additional file 2) in urea-containing growth medium. Previous work has showed that the parental strain (DSM1313 *Δhpt*) also had low urease expression in urea-containing fermentation medium, but that deletion of the GS gene caused significant upregulation of urease genes [47]. Consistent with higher expression of the urease genes, higher terminal pH was also previously observed for the GS-mutant fermentations compared to the parental strain fermentations [47]. It remains to be determined if higher urease expression increases *C. thermocellum*’s intracellular pH and enables steady-state growth at pH values below 6.25 with a dilution rate of 0.1 h^−1^. The GS mutant had less residual cellobiose and glucose in unbuffered medium than LL1210 and other nitrogen assimilation mutant strains tested (Additional file 4). Therefore, deletion of the GS in LL1210 might also improve substrate utilization and reduce or eliminate the need for buffering fermentation media.

## Conclusions

*Clostridium thermocellum* LL1210 can grow under slightly acidic conditions, similar to limits reported for related *C. thermocellum* strains. In nature, *C. thermocellum* may be exposed to acidic pH values, and if it is, metabolism will slow or the bacterium could sporulate, which are not aligned with industrial processes. This foundational study provides a detailed characterization of a relatively acid-intolerant bacterium and provides genetic targets for strain improvement. Future studies should examine adding gene functions used by more acid-tolerant bacteria for improved pH homeostasis at acidic pH values.

## Methods

### Bacterial strains, media and growth

*Clostridium thermocellum* strains DSM1313 ∆*hpt* [10], LL1210 [18], M1726 (LL376) [48], M1725 (LL375) [48], and AG1329 [47] were used in this study. The GLDH (Clo1313_1847), GOGAT (Clo1313_2032-6), GS–GOGAT (Clo1313_2031-6), and a *nifH* (Clo1313_2339) strain were deleted in a *C. thermocellum* DSM1313 ∆*hpt* background to create AG1327, AG1328, AG2068, and AG2078, respectively. Mutants were constructed using the procedures described in detail [49, 50]. Strains are available upon request. Primers used to construct strain AG2078 and other strains used in this study as well as SNPs identified from genome sequencing are provided in Additional file 8. Strains were stored frozen at − 80 °C in a modified version of DSM 122 medium, referred to as CTFUD [9], that contains 50 mM of MOPS (morpholinepropanesulfonic acid) and 10 mM of sodium citrate. Actively growing cultures were transferred twice from freezer stocks prior to batch fermentation experiments and inocula were 2% of the final volume. Cells were routinely cultured at 55 °C in either 50 ml (160 ml serum bottles) or 10 ml (26 ml Balch tubes) of MTC medium as described earlier [51], except that MOPS was omitted in this study. The LL1210 strain was also cultured in 1 ml volumes in a 48-well plate reader (Biotek Eon, Winooski, VT, USA) inside a Coy anaerobic chamber kept at 55 °C with growth data collected at OD_600 nm_ every 15 min. Growth in MTC-containing 100 μM of spermine (AC132750010, Fisher Scientific, Hampton, NH, USA), spermidine (S0266, Sigma-Aldrich, St. Louis, MO, USA), putrescine (AC112120250, Fisher Scientific) or arginine (A5006, Sigma-Aldrich) was assessed in plate reader assays.

Strain LL1210 was grown at 55 °C in duplicate 750 ml (total vessel capacity 1.3 L) chemostat cultures using water-jacketed BioFlo110 bioreactors (New Brunswick Scientific, Edison, NJ, USA), essentially as described previously in another *C. thermocellum* chemostat study [52], except that the medium contained 3 g/L of cellobiose as the carbon source. Briefly, the MTC medium was MOPS free and fed at a dilution rate of 0.1 h^−1^, except where noted, for 1058 h. Temperature, pH, and agitation speed were monitored and controlled during fermentation using the BioCommand software (version 2.62). Gel-reference pH electrodes Mettler-Toledo (Woburn, MA) calibration, accuracy checks and pH control were maintained as described earlier [23], except that 1 N HCl and 3 N KOH were used for acid and base additions, respectively. Recalibration of the bioreactor probes was performed at 546 h after pH drifted by 0.08 and 0.12. Samples for omics were taken in 50 ml volumes; then harvested and stored as described in [52]. For transcriptomics, two samples were taken from different chemostats at pH 6.48 and 6.12, and for pH values 6.98 and 6.24 biological duplicates and two technical replicates separated by at least one change of vessel medium were collected. For intracellular metabolomics, samples were taken immediately after transcriptomic samples, and additional biological duplicate samples were taken at pH 5.8 for metabolomics only. Culture turbidity was measured taking optical density readings at 600 nm using a Genesys 20 spectrophotometer (Thermo Fisher Scientific Inc., Waltham, MA, USA).

Cells were enumerated using a modified version of the direct cell counting procedure described in [53]. Briefly, 1 ml of culture samples were preserved for up to 1 week in 0.025 mg/ml (final concentration) paraformaldehyde (Sigma-Aldrich,). Cells were diluted 1:1000 with 0.9% (m/v) saline solution and stained with 25 nM 4′,6-diamidino-2-phenylindole (DAPI) (final concentration), immobilized to a 1.5 cm diameter section of a black MilliQ filter, and then imaged with a Zeiss Axio Imager.M1 with a Plan Apochromat 63×/1.4 Oil Immersion objective and X-Cite Series 120 Exfo fluorescence power source (Carl Zeiss, Germany). 8-bit images were imported into ImageJ (http://imagej.nih.gov/ij/) for manual or automated counting. Manual counting was performed with the cell counter plugin, and automated counting was performed with JAVA scripts (Additional file 1) which assumed cells were visible at a % Area (saturation threshold) of 1% (± 0.1%) with a particle size greater than 5 pixels after binary images had been processed with Watershed. Spherical morphologies were assumed to have a particle size range of 20–200 pixels and circularity of 0.85–1.00 for conservative estimates, and a particle size range of 50–100 pixels and circularity of 0.95–1.00 for liberal estimates. Automated parameters were validated with manual counts of 24 images. Stepwise calculations of standard deviation of progressively more sample counts were performed to ensure the count variance plateaued. Highly refractive mature spores were also observed by light microscopy after heat fixing to 20 μl of stationary cells to glass slides.

### Substrate and metabolite analyses

Extracellular fermentation product samples for high-performance liquid chromatography (HPLC) were collected and processed as described in [51]. HPLC data were generated using a Shimadzu Prominence LC-20A Series system (Shimadzu Scientific Instruments, Columbia, MD) fitted with a refractive index detector (model RID-20A) and an Aminex HPX 87H HPLC column (300 × 7.8 mm) (Bio-Rad, Hercules, Dallas, TX, USA) against known standards for cellobiose, glucose, ethanol, acetate, lactate, and formate. Intracellular metabolite fold changes and select metabolite concentrations were determined using a 5975C inert XL gas chromatography–mass spectrometer (Agilent, Santa Clara, CA) [22]. Extracellular amino acid concentrations were quantified by an Aracus Amino Acid Analyzer (membraPure, Berlin, Germany) [47]. Two-tailed paired *T* tests were performed to determine if amino acid concentrations were significantly different. Homoscedasticity was confirmed by performing Breusch–Pagan and Abridged White’s tests prior to performing *T* tests.

### RNA isolation, cDNA library preparation, sequencing and RNA-seq analysis

Total RNA was isolated from 50 ml of chemostat sample using the procedures described in [54], and quantity and quality were determined using a Nanodrop instrument (ThermoFisher Scientific Waltham, MA). Depletion, library preparation and sequencing were completed at the Joint Genome Institute (JGI, Walnut Creek, CA). In short, 100 ng of total RNA was depleted of ribosomal material using a Ribo-Zero (TM) rRNA Removal Kit (Epicentre). The rRNA depleted RNA was fragmented and reversed transcribed using random hexamers and SSII (Invitrogen) followed by second strand synthesis. Stranded cDNA libraries were generated using the Illumina Truseq Stranded RNA LT kit, following the manufactures protocols, using ten cycles of PCR. qPCR was used to determine the concentration of the libraries. Libraries were pooled and sequenced on an Illumina HiSeq 2500 platform with version 2 chemistry in a 2 × 151 bp configuration (JGI, Walnut Creek, CA; Project ID 503106). Fastq files were verified for integrity by checksum and read quality checked using FastQC [55]. RNAseq reads were trimmed using Trimmomatic MAXINFO method with target length and strictness parameters set to 40 and 0.8, respectively [56]. Reads were mapped to *C. thermocellum* DSM1313 (NC_017304, last modified 2015/07/30) using Bowtie2 with the same parameters as the very-sensitive preset option expect the number of mismatches was set to 1 [57, 58], and reads were counted with HTSeq [59]. Principle component analysis (PCA) was used to check for sample variation using JMP Genomics 8 (SAS Institute, Cary, NC, USA). Differential expression analysis was performed with DESeq 2 [60]. Genes were considered significantly differential expressed when their adjusted *p* values were less than or equal to 0.05 and log2-fold change values were ≥ 1 or ≤ 1. Raw RNA-seq data have been deposited in NCBI SRA under project number PRJNA395926. PCA plots, raw counts, RPKM normalized gene counts, and differential expression are provided in Additional file 2. Genes found to be differentially expressed at pH 6.48, 6.23, and pH 6.12 compared to pH 6.98 were clustered by their expression patterns using the *K*-means cluster procedure in JMP Genomics 8. Fisher’s exact test was then performed on 7 of the 9 *K*-means clusters to identify enriched GO terms. Prior to this analysis, GO terms were identified for the entire *C. thermocellum* DSM1313 genome using the standard workflow in Blast2GO 4.1.5 [61].

## Additional files

**Additional file 1.** Data logs, substrate and product concentrations, internal and external pH readings, and media formulation for chemostat reactor cultures of *C. thermocellum* LL1210.

**Additional file 2.** Raw and processed read counts, alignment statistics, log2-fold changes in the gene expression, and *K*-means clusters and GO enrichment of differentially expressed genes from samples taken from *C. thermocellum* LL1210 cultured in chemostats at pH values 6.98, 6.48, pH 6.24, and pH 6.12 (washout conditions). Gene expression at pH 6.98 was used as a reference for differential expression at lower pH values.

**Additional file 3: Figure S1.** Average optical density at wave-length 600nm (A and C) and average terminal pH (B and D) of *C. thermocellum* LL1210 cultured in 48-well plates. OD_600nm_ readings were taken automatically every 15 min in a microplate spectrophotometer (Biotek Eon, Winooski, VT) kept in an anaerobic chamber. Only 3-h time points are shown. Nine hundred microliters of inoculated medium was mixed with 100 μl of uninoculated medium supplemented with spermine, spermidine, or putrescine (polyamines), or arginine (polyamine precursor) so that the final concentration was 100 μM. Initial culture pH was 7.00 (A and B) or 6.75 (C and D). Averages were calculated from at least three biological replicates. Error bars indicate standard deviation and are colored the same as the amendments in the legend. **Table S1.** Average and standard deviation of maximum and terminal optical densities (600nm) and specific growth rate of *C. thermocellum* LL1210 cultured in media with and without amendments and having initial pHs of 7.00 and 6.75.

**Additional file 4: Figure S2.** Average growth (A), terminal pH (B), and remaining substrates and products at the end of *C. thermocellum*-mutant strain fermentations of cellobiose in MOPS-free carbon-replete medium (C). Averages were computed with data from four biological replicates. Error bars in each graph indicate standard deviation. Some error bars are too small to see. Deletion mutants are designated as LL1210 (hydrogenase maturation protein, lactate dehydrogenase, pyruvate formate lyase, phosphotransacetylase and acetate kinase), GLDH (glutamate dehydrogenase), GS (glutamine synthetase), GOGAT (glutamate synthase), GS-GOGAT (both), and NifH (nitrogenase iron protein). The parental strain designated DSM1313 has a deletion in the hypoxanthine phosphoribosyltransferase.

**Additional file 5: Figure S3.** Differential expression of genes found in Clostridia sporulation cascades. pro-σ^E^ processing protease is a stage III sporulation factor. BofA is an inhibitor of the stage IV pro-σ^K^ processing protease SpoIVFB. **Table S2.** Percentage of spherical morphologies 144 and 216 h after inoculation. **Figure S4.** Substrates and products (A) and the pH (B) after 144 and 216 h of *C. thermocellum*-mutant fermentations on MOPS-free carbon-replete medium starting with an initial pH of 6.75. Significant differences at α = 0.001 for comparisons with DSM1313 (∆*hpt*) are indicated with a “*” and comparisons with DSM1313 (∆*hpt*) and LL1210 are indicated with “**”. Averages were calculated with six biological replicates. Error bars indicate standard deviation.

**Additional file 6: Table S3.** Fold changes of intercellular metabolite there were significantly higher or lower in concentration at growth-limiting pHs.

**Additional file 7.** Extracellular amino acid concentrations in media from *C. thermocellum* LL1210 chemostats that were sampled when pH values were pH 6.48, pH 6.24, pH 6.12, and below. Demonstrations of data homoscedasticity for *T* tests.

**Additional file 8: Table S4.** Strains and primers used in this study.
