# Supplementary material for: Clostridium thermocellum LL1210 pH homeostasis mechanisms informed by transcriptomics and metabolomics
Source: Biotechnol Biofuels. 2018 Apr 5;11:98. doi: 10.1186/s13068-018-1095-y (PMC5887222; doi:10.1186/s13068-018-1095-y)
Supplement: Supplementary file 3 — Additional file 3: Figure S1. Average optical density at wave-length 600nm (A and C) and average terminal pH (B and D) of C. thermocellum LL1210 cultured in 48-well plates. OD600nm readings were taken automatically every 15 min in a microplate spectrophotometer (Biotek Eon, Winooski, VT) kept in an anaerobic chamber. Only 3-h time points are shown. Nine hundred microliters of inoculated medium was mixed with 100 μl of uninoculated medium supplemented with spermine, spermidine, or putrescine (polyamines), or arginine (polyamine precursor) so that the final concentration was 100 μM. Initial culture pH was 7.00 (A and B) or 6.75 (C and D). Averages were calculated from at least three biological replicates. Error bars indicate standard deviation and are colored the same as the amendments in the legend. Table S1. Average and standard deviation of maximum and terminal optical densities (600nm) and specific growth rate of C. thermocellum LL1210 cultured in media with and without amendments and having initial pHs of 7.00 and 6.75. [file 13068_2018_1095_MOESM3_ESM.docx]

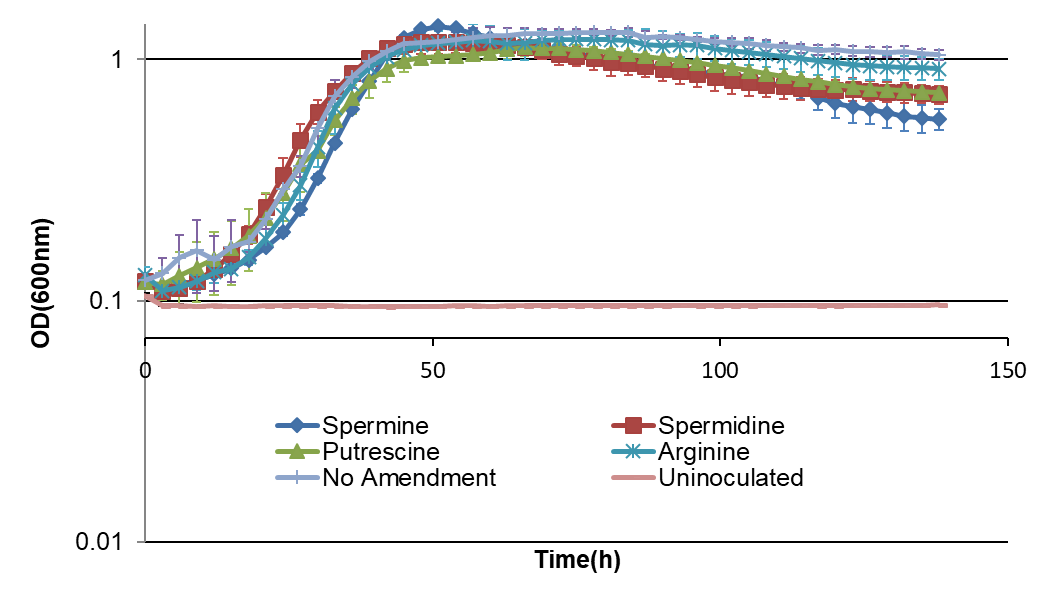

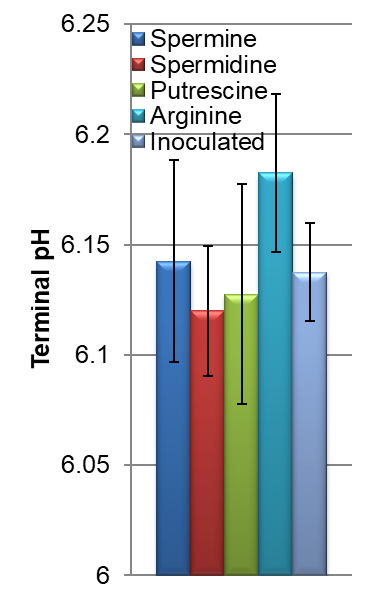


A

B


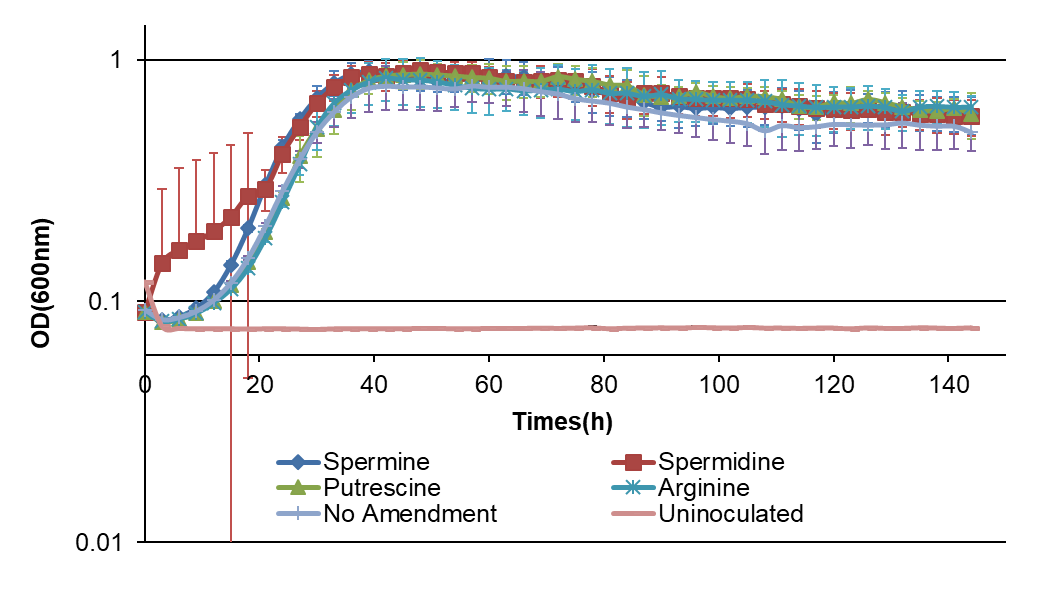

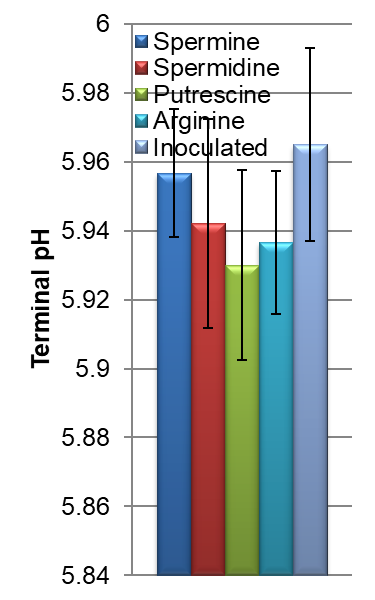


C

D

Additional file 3: Figure S1. Average optical density at wave-length 600nm (A and C) and average terminal pH (B and D) of *C. thermocellum* LL1210 cultured in 48-well plates. OD_600nm_ readings were taken automatically every 15 min in a microplate spectrophotometer (Biotek Eon, Winooski, VT) kept in an anaerobic chamber. Only 3 h time points are shown. Nine hundred microliters of inoculated medium was mixed with 100 μl of uninoculated medium supplemented with spermine, spermidine, or putrescine (polyamines), or arginine (polyamine precursor) so that the final concentration was 100 μM. Initial culture pH was 7.00 (A and B) or 6.75 (C and D). Averages were calculated from at least three biological replicates. Error bars indicate standard deviation and are colored the same as the amendments in the legend.

| Additional file 3: Table S1. Average and standard deviation of maximum and terminal optical densities (600nm) and specific growth rate of *C. thermocellum* LL1210 cultured in media with and without amendments and having initial pHs of 7.00 and 6.75. | | | | | | | | | | |  |  |  |  |
| --- | --- | --- | --- | --- | --- | --- | --- | --- | --- | --- | --- | --- | --- | --- |
| Average (Standard Deviation) | Spermine | | Spermidine | | Putrescine | | Arginine | | No Treatment | |  |  |  |  |
|  |  | |  | |  | |  | |  | |  |  |  |  |
|  | 7.00 6.75 | | 7.00 6.75 | | 7.00 6.75 | | 7.00 6.75 | | 7.00 6.75 | |  |  |  |  |
| OD_max_ | 1.373 (0.024)^a^ | 0.921 (0.110) | 1.205 (0.108)^a^ | 0.932 (0.093) | 1.138 (0.074)^a,b^ | 0.910 (0.096) | 1.280 (0.154)^a^ | 0.877 (0.167) | 1.339 (0.048)^a^ | 0.814 (0.147) |  |  |  |  |
| OD_term_ | 0.564 (0.057)^c^ | 0.594 (0.107) | 0.716 (0.061)^c^ | 0.585 (0.099) | 0.728 (0.020)^c^ | 0.599 (0.127) | 0.912 (0.092)^c^ | 0.640 (0.067)^d,e^ | 1.044 (0.047) | 0.502 (0.081)^d^ |  |  |  |  |
| h ^-1^ | 0.111 (0.005)^f^ | 0.108 (0.006) | 0.110 (0.011) | 0.108 (0.010) | 0.098 (0.005) | 0.115 (0.022) | 0.106 (.004)^f^ | 0.116 (0.013) | 0.098 (0.004) | 0.108 (0.011) |  |  |  |  |
| ^a^Average maximum OD was significantly higher (α < 0.05) when the initial pH of the medium was 7.00 compared to 6.75 | | | | | | | | | | | | |  |  |
| ^b^Average maximum OD was significantly higher (α < 0.05) with amendment than without amendment at pH 7.00 | | | | | | | | | | | |  |  |  |
| ^c^Average terminal OD was significantly higher (α < 0.05) with amendment than without amendment at pH 7.00 | | | | | | | | | | | |  |  |  |
| ^d^Average terminal OD was significantly higher (α < 0.05) when the initial pH of the medium was 7.00 compared to 6.75 | | | | | | | | | | | | |  |  |
| ^e^Average terminal OD was significantly higher (α < 0.05) with amendment than without amendment at pH 6.75  ^f^Average specific growth rate was significantly higher (α < 0.05) with amendment than without amendment at pH 6.75 | | | | | | | | | | | |  |  |  |
