# Supplementary material for: Clostridium thermocellum LL1210 pH homeostasis mechanisms informed by transcriptomics and metabolomics
Source: Biotechnol Biofuels. 2018 Apr 5;11:98. doi: 10.1186/s13068-018-1095-y (PMC5887222; doi:10.1186/s13068-018-1095-y)
Supplement: Supplementary file 4 — Additional file 4: Figure S2. Average growth (A), terminal pH (B), and remaining substrates and products at the end of C. thermocellum-mutant strain fermentations of cellobiose in MOPS-free carbon-replete medium (C). Averages were computed with data from four biological replicates. Error bars in each graph indicate standard deviation. Some error bars are too small to see. Deletion mutants are designated as LL1210 (hydrogenase maturation protein, lactate dehydrogenase, pyruvate formate lyase, phosphotransacetylase and acetate kinase), GLDH (glutamate dehydrogenase), GS (glutamine synthetase), GOGAT (glutamate synthase), GS-GOGAT (both), and NifH (nitrogenase iron protein). The parental strain designated DSM1313 has a deletion in the hypoxanthine phosphoribosyltransferase. [file 13068_2018_1095_MOESM4_ESM.docx]

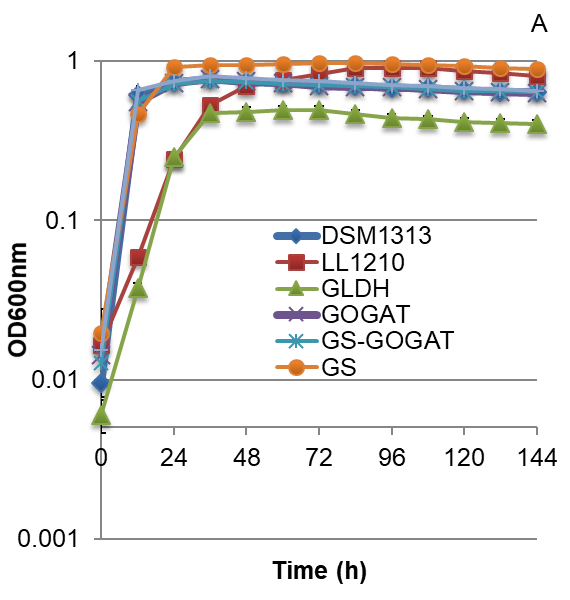

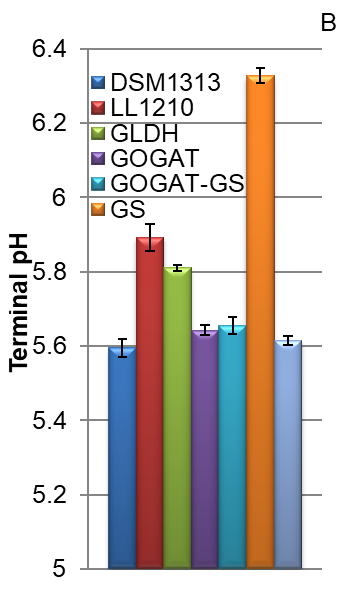


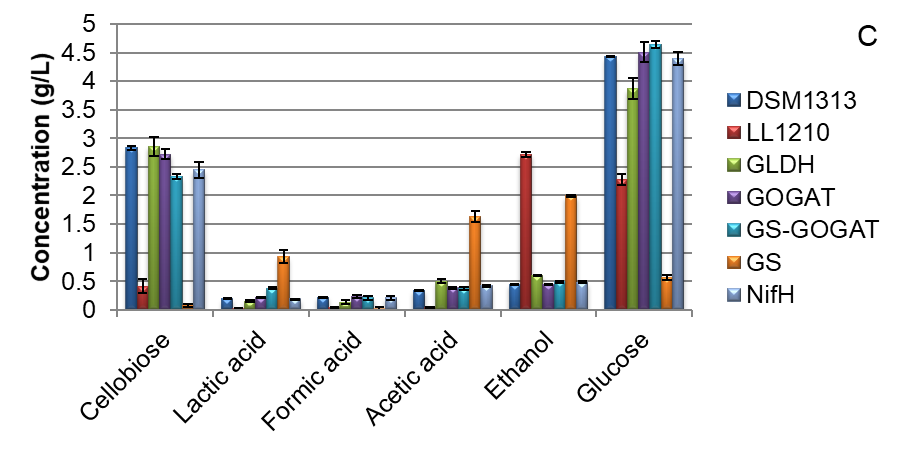


Additional file 4: Figure S2. Average growth (A), terminal pH (B), and remaining substrates and products at the end of *C. thermocellum* mutant strain fermentations of cellobiose in MOPS-free carbon-replete medium (C). Averages were computed with data from four biological replicates. Error bars in each graph indicate standard deviation. Some error bars are too small to see. Deletion mutants are designated as LL1210 (hydrogenase maturation protein, lactate dehydrogenase, pyruvate formate lyase, phosphotransacetylase and acetate kinase), GLDH (glutamate dehydrogenase), GS (glutamine synthetase), GOGAT (glutamate synthase), GS-GOGAT (both), and NifH (nitrogenase iron protein). The parental strain designated DSM1313 has a deletion in the hypoxanthine phosphoribosyltransferase.
