# Supplementary material for: Clostridium thermocellum LL1210 pH homeostasis mechanisms informed by transcriptomics and metabolomics
Source: Biotechnol Biofuels. 2018 Apr 5;11:98. doi: 10.1186/s13068-018-1095-y (PMC5887222; doi:10.1186/s13068-018-1095-y)
Supplement: Supplementary file 5 — Additional file 5: Figure S3. Differential expression of genes found in Clostridia sporulation cascades. pro-σE processing protease is a stage III sporulation factor. BofA is an inhibitor of the stage IV pro-σK processing protease SpoIVFB. Table S2. Percentage of spherical morphologies 144 and 216 h after inoculation. Figure S4. Substrates and products (A) and the pH (B) after 144 and 216 h of C. thermocellum-mutant fermentations on MOPS-free carbon-replete medium starting with an initial pH of 6.75. Significant differences at α = 0.001 for comparisons with DSM1313 (∆hpt) are indicated with a “*” and comparisons with DSM1313 (∆hpt) and LL1210 are indicated with “**”. Averages were calculated with six biological replicates. Error bars indicate standard deviation. [file 13068_2018_1095_MOESM5_ESM.docx]

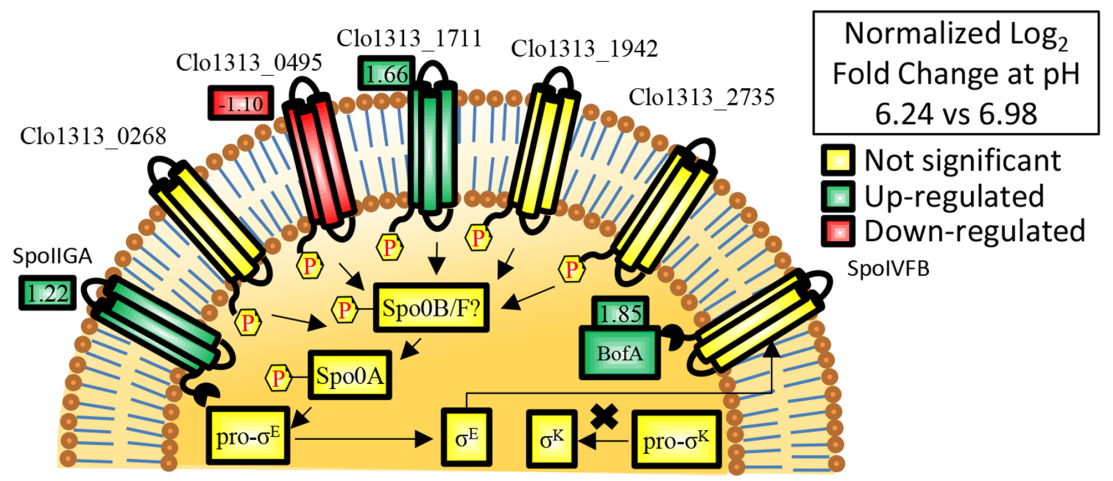


Additional file 5: Figure S3. Differential expression of genes found in Clostridia sporulation cascades. pro-σ^E^ processing protease is a stage III sporulation factor. BofA is an inhibitor of the stage IV pro-σ^K^ processing protease SpoIVFB.

| Additional file 5: Table S2. Percentage of spherical morphologies 144 and 216 h after inoculation | | | | |
| --- | --- | --- | --- | --- |
| Strain | Conservative | Liberal | Conservative | Liberal |
| DSM1313 *∆hpt* | 0% | 2% | 1% | 8% |
| M1726 | 1% | 4% | 0% | 0% |
| LL1210 | 1% | 5% | 1% | 5% |
| M1725 | 0% | 1% | 0% | 2% |


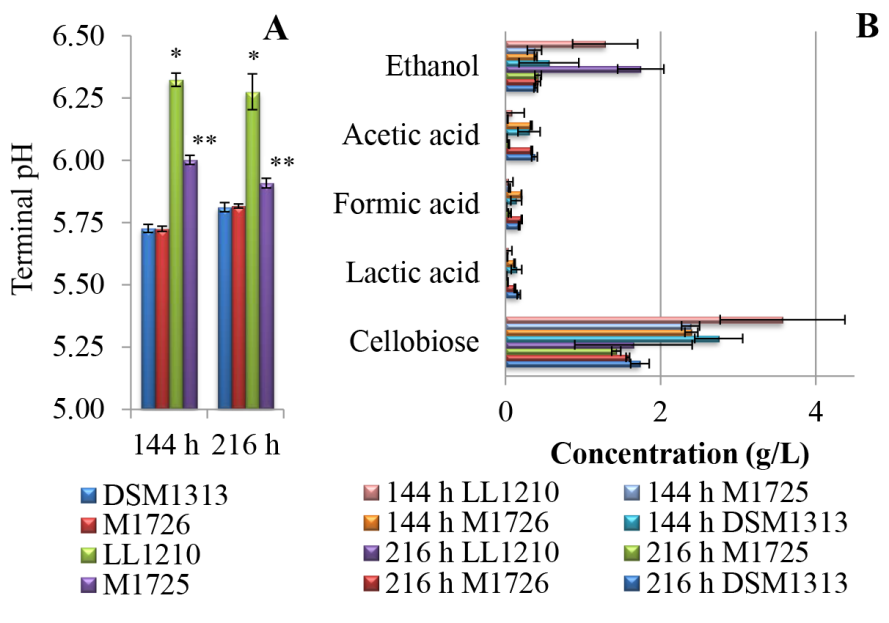


Additional file 5: Figure S4. Substrates and products (A) and the pH (B) after 144 and 216 h of *C. thermocellum* mutant fermentations on MOPS-free carbon-replete medium starting with an initial pH of 6.75. Significant differences at α = 0.001 for comparisons with DSM1313 (∆*hpt*) are indicated with a "*" and comparisons with DSM1313 (∆*hpt*) and LL1210 are indicated with "**". Averages were calculated with six biological replicates. Error bars indicate standard deviation.
