# Supplementary material for: Clostridium thermocellum LL1210 pH homeostasis mechanisms informed by transcriptomics and metabolomics
Source: Biotechnol Biofuels. 2018 Apr 5;11:98. doi: 10.1186/s13068-018-1095-y (PMC5887222; doi:10.1186/s13068-018-1095-y)
Supplement: Supplementary file 8 — Additional file 8: Table S4. Strains and primers used in this study. [file 13068_2018_1095_MOESM8_ESM.docx]

Additional file 8: Table S4. Strains and primers used in this study

| Strains used in this study | | | |
| --- | --- | --- | --- |
| Strain | Description | GOLD Project ID | Reference |
| DSM1313 ∆*hpt* |  |  | Biswas *et al.* 2014 |
| M1726 (LL376) | DSM1313 ∆*hpt* | Gp0090720 | Dash *et al.* 2017 |
| M1725 (LL375) | DSM1313 ∆*hpt* | Gp0090719 | Dash *et al.* 2017 |
| LL1210 | DSM1313 ∆*hpt* | Gp0110570 | Tian *et al.* 2016 |
| AG2078 | DSM1313 ∆*hpt* ∆*nifH* | Gp0177669 | This study |
| AG1329 | DSM1313 ∆*hpt* ∆Clo1313_2031 | Gp0113506 | Rydzak *et al.* 2017 |
| AG1327 | DSM1313 ∆*hpt* ∆ Clo1313_1847 | Gp0113504 | Request from A. Guss |
| AG1328 | DSM1313 ∆*hpt* ∆ Clo1313_2032-36 | Gp0113505 | Request from A. Guss |
| AG2068 | DSM1313 ∆*hpt* ∆ Clo1313_2031-36 | Gp0147172 | Request from A. Guss |

| Primers used to verify gene deletions of strains used in this study | | |
| --- | --- | --- |
| Primer | Target | Sequence |
| OA27 | *pfl* | ATACTTGATTATTATGAGCGCGG |
| OA11 | *pfl* | ATTCTCCTGGTTAAGCCTTGTAA |
| OA20 | *ech* | AGCGCGGATATATGATGGC |
| OA21 | *ech* | CGTAAGGCGAAATTGAAAAGGAA |
| OA34 | *ldh* | CTTGGCTTCATTGCTGTAAGATAC |
| OA35 | *ldh* | ACCGCTGGAACATTAACAGATT |
| OA39 | *pta-ack* | CGCAGGAGATAGTAAGGGAAGA |
| OA38 | *pta-ack* | AATCCAAAACCTCCCATTCTTT |
| OA5 | *hydG* | GCTGTAAGTCTTCGGTGAGAGTT |
| OA6 | *hydG* | CTCACTTTTGTAGAATCCACACCT |
| Spo0A1_F | *spo0A1* | AAAATGTTGAAATGGCCAATGC |
| Spo0A1_R | *spo0A1* | AACAATCACAAGACTTGCGCT |
| Spo0A2_F | *spo0A2* | GCAGTTAAAGAAGATGGGTGCG |
| Spo0A2_R | *spo0A2* | GAATTGCTTGACTTTCCGCGA |

| *C. thermocellum* AG2078 SNPs identified by genome sequencing | | | | | | | | | | | |
| --- | --- | --- | --- | --- | --- | --- | --- | --- | --- | --- | --- |
| #Software: 1.4.5 (commit 251f983) | | | |  |  |  |  |  |  |  |  |
| #Command: breakdancer_max 1517_1127198/bwa_dir/1517_1127198.bd.config | | | | | | | |  |  |  |  |
| #Library Statistics: | |  |  |  |  |  |  |  |  |  |  |
| #1517_1127198/bwa_dir/1517_1127198.bam | mean:378.96 | std:95.61 | uppercutoff:575 | lowercutoff:233 | readlen:100 | library:BNGNT | reflen:3574929 | seqcov:135.295 | phycov:256.358 | 1.509722 | 2:165649 |
| #Chr1 | Pos1 | Orientation1 | Chr2 | Pos2 | Orientation2 | Type | Size | Score | num_Reads | num_Reads_lib | 1517_1127198.bam |
| CP002416 | 448268 | 101+10- | CP002416 | 448756 | 157+117- | INV | 70 | 99 | 91 | 1517_1127198/bwa_dir/1517_1127198.bam\|91 | 0.28 |
| CP002416 | 448087 | 17+7- | CP002416 | 448756 | 157+117- | INV | 221 | 99 | 14 | 1517_1127198/bwa_dir/1517_1127198.bam\|14 | 0.63 |
| CP002416 | 448312 | 19+4- | CP002416 | 448756 | 157+117- | INV | -132 | 99 | 18 | 1517_1127198/bwa_dir/1517_1127198.bam\|18 | 0.14 |
| CP002416 | 448756 | 157+117- | CP002416 | 448965 | 13+84- | INV | 7 | 99 | 77 | 1517_1127198/bwa_dir/1517_1127198.bam\|77 | 0.15 |
| CP002416 | 448756 | 157+117- | CP002416 | 449018 | 6+13- | INV | 241 | 99 | 11 | 1517_1127198/bwa_dir/1517_1127198.bam\|11 | 0.98 |
| CP002416 | 706831 | 19+47- | CP002416 | 707405 | 99+77- | ITX | -272 | 99 | 36 | 1517_1127198/bwa_dir/1517_1127198.bam\|36 | 0.82 |
| CP002416 | 707083 | 99+77- | CP002416 | 707703 | 77+18- | ITX | -224 | 99 | 42 | 1517_1127198/bwa_dir/1517_1127198.bam\|42 | 0.63 |
| CP002416 | 2746997 | 186+22- | CP002416 | 2747782 | 46+202- | DEL | 880 | 99 | 170 | 1517_1127198/bwa_dir/1517_1127198.bam\|170 | 0.37 |
| CP002416 | 2789384 | 35+198- | CP002416 | 3242533 | 20+180- | INV | 452730 | 99 | 144 | 1517_1127198/bwa_dir/1517_1127198.bam\|144 | 2.01 |
| CP002416 | 2789520 | 19+42- | CP002416 | 3242533 | 20+180- | INV | 452486 | 99 | 21 | 1517_1127198/bwa_dir/1517_1127198.bam\|21 | 2.01 |
| CP002416 | 2790362 | 226+56- | CP002416 | 3242247 | 183+22- | INV | 451517 | 99 | 168 | 1517_1127198/bwa_dir/1517_1127198.bam\|168 | 2.01 |
| CP002416 | 3435331 | 148+18- | CP002416 | 3435740 | 20+186- | DEL | 457 | 99 | 113 | 1517_1127198/bwa_dir/1517_1127198.bam\|113 | 0.01 |
| CP002416 | 1 | 26+157- | CP002416 | 3561690 | 171+35- | ITX | 3560835 | 99 | 143 | 1517_1127198/bwa_dir/1517_1127198.bam\|143 | 2.01 |
| CP002416 | 2747322 | 2+34- | pDGO-63 | 3261 | 9+0- | CTX | -378 | 99 | 7 | 1517_1127198/bwa_dir/1517_1127198.bam\|7 | |
| CP002416 | 2747322 | 2+34- | pDGO-63 | 3293 | 23+7- | CTX | -378 | 99 | 17 | 1517_1127198/bwa_dir/1517_1127198.bam\|17 | |
| CP002416 | 2747322 | 2+34- | pDGO-63 | 3391 | 13+3- | CTX | -378 | 99 | 4 | 1517_1127198/bwa_dir/1517_1127198.bam\|4 | |
| CP002416 | 2748217 | 46+14- | pDGO-63 | 2249 | 0+8- | CTX | -378 | 99 | 5 | 1517_1127198/bwa_dir/1517_1127198.bam\|5 | |
| CP002416 | 2748217 | 46+14- | pDGO-63 | 2294 | 3+8- | CTX | -378 | 99 | 7 | 1517_1127198/bwa_dir/1517_1127198.bam\|7 | |
